# Resveratrol Protects against TNF-α-Induced Injury in Human Umbilical Endothelial Cells through Promoting Sirtuin-1-Induced Repression of NF-KB and p38 MAPK

**DOI:** 10.1371/journal.pone.0147034

**Published:** 2016-01-22

**Authors:** Wei Pan, Huizhen Yu, Shujie Huang, Pengli Zhu

**Affiliations:** 1 Provincial Clinical Medical College, Fujian Medical University, Fuzhou, China; 2 Department of Geriatrics, Fujian Provincial Hospital Key Laboratory of Geriatrics, Fujian Medical University, Fuzhou, China; 3 Fujian Institute of Clinical Geriatrics, Fuzhou, China; University of Missouri-Kansas City, UNITED STATES

## Abstract

Inflammation and reactive oxygen species (ROS) play important roles in the pathogenesis of atherosclerosis. Resveratrol has been shown to possess anti-inflammatory and antioxidative stress activities, but the underlying mechanisms are not fully understood. In the present study, we investigated the molecular basis associated with the protective effects of resveratrol on tumor necrosis factor-alpha (TNF-α)-induced injury in human umbilical endothelial cells (HUVECs) using a variety of approaches including a cell viability assay, reverse transcription and quantitative polymerase chain reaction, western blot, and immunofluorescence staining. We showed that TNF-α induced CD40 expression and ROS production in cultured HUVECs, which were attenuated by resveratrol treatment. Also, resveratrol increased the expression of sirtuin 1 (SIRT1); and repression of SIRT1 by small-interfering RNA (siRNA) and the SIRT1 inhibitor Ex527 reduced the inhibitory effects of resveratrol on CD40 expression and ROS generation. In addition, resveratrol downregulated the levels of p65 and phospho-p38 MAPK, but this inhibitory effect was attenuated by the suppression of SIRT1 activity. Moreover, the p38 MAPK inhibitor SD203580 and the nuclear factor (NF)-κB inhibitor pyrrolidine dithiocarbamate (PDTC) achieved similar repressive effects as resveratrol on TNF-α-induced ROS generation and CD40 expression. Thus, our study provides a mechanistic link between resveratrol and the activation of SIRT1, the latter of which is involved in resveratrol-mediated repression of the p38 MAPK/NF-κB pathway and ROS production in TNF-α-treated HUVECs.

## Introduction

The pathogenesis of atherosclerosis is a complex pathological process that involves an ongoing inflammatory response in blood vessels, which mediates all stages of atherosclerosis including initiation and progression [1]. Recently, CD40, the B cell surface antigen, has been reported to be upstream of the cytokine signaling network, which plays an important role in inflammation. Activation of CD40 by proinflammatory cytokines such as CD40L (CD154) has been shown to augment the expression of matrix metalloproteinases, procoagulant tissue factor, chemokines, and cytokines[2], which regulate various inflammatory responses in the process of atherosclerosis[3]. Therefore, strategies designed to suppress CD40/CD40L expression may attenuate inflammation, which will eventually offer benefits during the progression of atherosclerosis.

Oxidative stress associated with reactive oxygen species (ROS), including superoxide (O^2-^), hydrogen peroxide (H_2_O_2_), hydroxide (OH^-^), and hypochlorite (OCl^-^), is also crucial to the pathogenesis of atherosclerosis. NADPH oxidase, enzymatic activation of cytochrome P450, xanthine oxidase, and inflammatory activities are considered to be the major sources of ROS [4]. A previous study has shown that the activation of CD40 inhibits endothelial cell migration by increasing ROS production [5], and another study has provided evidence that ROS mediate a key initiating step in the development of atherosclerosis [6]. These findings point to a role for CD40 in the regulation of ROS generation.

Considering the major roles of inflammation and oxidative responses in the pathogenesis of atherosclerosis, the inflammatory and oxidative pathways have been the therapeutic targets for atherosclerosis. Studies have shown that light to moderate consumption of red wine reduces the risk of developing cardiovascular events owing to the polyphenolic component resveratrol[7]. Indeed, resveratrol has many interesting properties, including the ability to reverse dyslipidemia and obesity, to inhibit hyperglycemia and hyperinsulinemia, and to protect endothelial function. Resveratrol also has been shown to act on multiple molecular targets important for cell differentiation and activation. For instance, Tsuruoka *et al*.[8] have discovered that resveratrol enhances mitochondrial function independent of peroxisome proliferator-activated receptor-gamma coactivator 1 alpha (PGC-1α), and Ido *et al*. [9] have found that resveratrol prevents oxidative stress-induced senescence and proliferative dysfunction by activating the 5ʹ-adenosine monophosphate-activated protein kinase (AMPK)–Forkhead Box O (FOXO) cascade. In addition, resveratrol has been shown to regulate variations in ion channels through modulating the phosphoinositide 3-kinase/Akt/endothelial nitric oxide synthase (eNOS) signaling pathway, thus decreasing left arterial fibrosis [10]. However, the effects of resveratrol on CD40 expression and ROS generation in endothelial cells are not fully understood.

It is known that a reasonable dose of resveratrol activates sirtuin 1 (SIRT1), a class III nicotinamide adenine dinucleotide (NAD)-dependent histone deacetylase [11]. SIRT1 deacetylates a variety of substrates to regulate the downstream signaling pathways. It has been demonstrated that SIRT1 suppresses the expression levels of inflammatory genes in a variety of cell types including adipocytes [12], endothelial cells, smooth muscle cells, and macrophages to protect the cardiovascular system from injury [13]. For instance, Harijith *et al*. [14] have found that the activation of inflammasomes is dampened by SIRT1. On the contrary, SIRT1 knockdown increases intercellular adhesion molecule-1 expression in endothelial cells [15], thus promoting inflammation. As such, resveratrol is considered to be a protective factor and participate in promoting anti-inflammatory responses through SIRT1-mediated signal pathways. However, the mechanisms underlying the specific role of SIRT1 in the CD40/CD40L axis and ROS production have not been reported in depth. Together, these findings prompted us to investigate the signaling pathways involved in mediating the resveratrol-directed anti-inflammatory reaction, and our findings will provide a molecular basis for the potential application of resveratrol in treating atherosclerosis.

## Materials and Methods

### Reagents

Resveratrol (SML0963) was obtained from Sigma-Aldrich (St. Louis, MO, USA). Tumor necrosis factor-alpha (TNF-α) (300-01A) was obtained from PeproTech (Rocky Hill, NJ, USA). Endothelial cell medium, fetal bovine serum, endothelial cell growth supplement, and penicillin/streptomycin solution (1001) were purchased from Sciencell Research Laboratories (Carlsbad, CA, USA). SB203580 (S1076), EX527 (S1541), and pyrrolidine dithiocarbamate (PDTC) (S1809) were purchased from Selleck Chemicals (Houston, TX, USA). Fluorescein isothiocyanate (FITC)-conjugated affiniPure goat anti-rabbit IgG (H+L) (111-095-144) was purchased from Jackson ImmunoResearch (West Grove, PA, USA). Rabbit anti-factor VIII-related antigen (BA0046) was purchased from Boster (Wuhan, China). Anti-CD40 (ab47021), SIRT1 (ab32441), p65 nuclear factor (NF)-κB (ab7970), phospho-p38 MAPK (ab4822) antibodies, BCA protein assay kit (ab102536) were obtained from Abcam (Abcam, Cambridge, MA, USA). Small interfering RNA (siRNA) specifically targeting SIRT1 and CD40 and a negative control siRNA (Nc-siRNA) were synthesized by GenePharma (Shanghai, China).

### Cell culture and immunocytochemistry

Our study was approved by the Ethics Committee of the Fujian Provincial Hospital (No. K2014-021-01), and all aspects of the study were in compliance with the Declaration of Helsinki. Each patient signed a consent form before providing human umbilical endothelial cells (HUVECs). Primary HUVEC cultures were established using the collagenase treatment method described previously by Baudin [16]. Briefly, human umbilical cords were collected within 6 h of delivery and were maintained on gelatin-coated T25 flasks in endothelial cell medium supplemented with 5% fetal bovine serum, 50 μg/mL endothelial cell growth supplement, and 1% penicillin/streptomycin solution at 37°C in a humidified atmosphere of 5% CO_2_/95% air. All experiments were performed on HUVECs at passage 3 to 5.

When the HUVECs were grown to 80–90% confluence, the cells were fixed in 4% paraformaldehyde for 10 min at room temperature, followed by three washes with phosphate-buffered saline (PBS). The fixed cells were permeabilized with 0.5% Triton X-100 for 10 min and blocked with 5% bovine serum albumin for 20 min. Then, the cells were incubated with the rabbit anti-factor VIII-related antigen (1:50 dilution) at 4°C overnight. Factor VIII was detected using a streptavidin biotin peroxidase complex kit and a 3,3’-diaminobenzidine kit. The stained cells were scored using an Olympus fluorescence microscope (Leica, Germany).

### Cell treatment

To induce an inflammatory response in the HUVECs, the cells were treated with different concentrations of TNF-α (0, 1, 10, or 20 μg/L) for 24 h. To explore the effect of resveratrol on TNF-α-induced cell injury, the cells were pretreated with different concentrations of resveratrol (0, 5, 10, and 20 μM) for 2 h in the presence or absence of TNF-α (10 μg/L) for 24 h. To explore the potential signaling pathways involved in the resveratrol-mediated cell protection, the cells were treated with Ex 527 (10 μM), SB203580 (10 μM), or PDTC (20 μM), respectively, in the absence or presence of resveratrol and TNF-α.

### Cell viability assay

Cell viability was assessed using a cell counting kit-8 (CCK-8) assay, according to the manufacturer’s recommendations. Briefly, cells (1 × 10^4^ cells/well) were plated onto 96-well plates and treated with drug-containing media for 24 h, CCK-8 (10 μL) was added to the media for a 2-h incubation, and the absorbance was measured at 450 nm with an enzyme-linked immunosorbent assay plate reader (Thermo, Boston, MA, USA).

### RNA interference

HUVECs were seeded on 6-well plates and then transiently transfected with synthesized small-interfering RNA (siRNA) targeting human SIRT1 and CD40 or a negative control siRNA (Nc-siRNA). The siRNA and Lipofectamine2000 transfection agent were separately diluted with serum-free medium, according to the manufacturer’s instructions. Then, the cells were transfected with siRNA-Lipofectamine complexes, and the silencing effect of the siRNA was evaluated by reverse transcription and quantitative polymerase chain reaction (RT-qPCR) as well as western blot at 48 h after transfection.

### Flow cytometry analysis of ROS

After each treatment, HUVECs (1 × 10^6^/mL) were incubated for 20 min at 37°C with or without the cell permeable fluorescent and chemiluminescent probe 2’-7’-dichlorodihydroofluorescein diacetate (DCFH-DA). After incubation, the cells were washed twice with sterile PBS, the ROS levels were detected by fluorescence-activated cell sorting (FACSCalibur, Beckton Dickinson, Oxford, UK), and the results were analyzed by CellQuest software (BD Biosciences, San Jose, CA, USA).

### Flow cytometry analysis of CD40 expression

Resveratrol and different signaling pathway inhibitors were incubated with cells with or without TNF-α in the media for 24 h. Thereafter, the cells were harvested and incubated with an anti-CD40 antibody for 1 h at 37°C, followed by incubation with human FITC-conjugated goat anti-rabbit IgG for 30 min at 4°C. An isotype-matched antibody was used as a control. The CD40 levels were detected by fluorescence-activated cell sorting (FACSCalibur, Beckton Dickinson, Oxford, UK), and the results were analyzed by CellQuest software (BD Biosciences, San Jose, CA, USA). The mean fluorescence intensity was calculated as described previously.

### Western blot analysis

Total cell lysates were extracted with ice-cold RIPA buffer and measured using a BCA protein assay kit. Cell extracts were fractionated by electrophoresis on 10% sodium dodecyl sulfate–polyacrylamide gels and transferred to polyvinylidene fluoride membranes for 2 h at 100 V. The membranes were subsequently blocked with 5% nonfat dry milk for 1 h at room temperature and overnight at 4°C with primary antibodies against human SIRT1 (1:200 dilution), CD40 (1:800 dilution), NF-κB p65 (diluted 1:500), p38 MAPK (diluted 1:800), or β-actin (1:400 dilution), respectively. The membranes were then incubated with horseradish peroxidase-conjugated anti-rabbit secondary antibody (1:5000 dilution) for 1 h at room temperature. Protein bands were visualized by the enhanced chemiluminescence (Gibco, Invitrogen Life Science, UK). Quantification of band intensity was carried out using Image J software (National Institutes of Health).

### RT-qPCR assay

Total RNA was isolated from HUVECs using Trizol reagent, according to the manufacturer’s protocol. The integrity of the RNA samples was checked by agarose gel electrophoresis. The total RNA was reverse-transcribed into cDNA using a reverse transcription kit, followed by quantitative PCR to detect the expression levels of SIRT1, CD40 and NF-κB. β-Actin was used as an internal control. The thermal cycling conditions were as follows: 30 s at 95°C for the activation of Taq DNA polymerase, followed by 40 cycles of amplification at 95°C for 15 s, 1 min at 60°C, and 1 min at 72°C. The reactions were run in triplicate. The specific oligos used in the real-time PCR were as follows: SIRT1 (forward): 5ʹ-GATTAGTAGGCGCTT GATGGT-3ʹ, SIRT1 (reverse): 5ʹ-TCTTCTAAACTTGGACTCGGCAT-3ʹ, CD40 (forward): 5ʹ-A CACTGCCACCAGCACAAATAC-3ʹ, CD40 (reverse): 5ʹ- GATAAAGACCAGCACCAAGAGGAT-3ʹ, NF-κB (forward): 5’ AGTTTGACGGTGAGCTGGTA 3’, NF-κB (reverse): 5’ GCCTCGGCCTGCCGCAAGCCT3’, β-actin (forward): 5ʹ-TGGCACCCAGCACAATGAA-3ʹ, β-actin (reverse): 5ʹ-CTAAGT CATAGTCCGCCTAGAAGCA-3ʹ. Gene expression values were calculated by the 2 ^-ΔΔCT^ relative expression method.

### NF-κB reporter gene assay

HUVECs (3 × 10^5^ cells/well) were transfected with siRNA targeting SIRT1 and the pGL4.32 [luc2P/NF-κB-RE/Hygro] vector using Lipofectamine. At 24 h post-transfection, the cells were pretreated with vehicle, resveratrol, or PDTC as indicated before TNF-α stimulation. The luciferase activity was determined by the luciferase assay kit.

### Immunofluorescence staining

Immunofluorescence staining was carried out when the cells reached 60–80% confluence. After each treatment mentioned above, the cells were fixed in 4% formaldehyde-PBS for 15 min at room temperature and then immersed in 0.3% TritonX-100-PBS for 30 min. Thereafter, the fixed and permeabilized HUVECs were incubated with the primary anti-SIRT1 antibody (1:150 dilution) at 4°C overnight, followed by another incubation with the polyclonal FITC-conjugated goat anti-rabbit IgG (1:50 dilution) for 1 h at room temperature as described previously [17]. Cellular nuclei were stained with DAPI (1:1000 dilution). Thirty cells from six different regions per group were randomly chosen and analyzed with a confocal scanning laser microscope equipped with an argon laser (Leica Lasertechnik, Heidelberg, Germany). The triple staining was analyzed using a Zeiss laser scanning microscope (LSM 510 META with an Axiovert 200 microscope). Images were taken using a Plan-Apochromat 63×/1.4 oil immersion objective. Fluorescence intensity was determined by computer-based image analysis system MetaView™ software by measuring the average pixel intensity within a fixed circled area placed over the relevant area of the cell [18].

### Statistical analysis

Data were collected from three independent experiments, each carried out in triplicate and expressed as the mean ± standard error of the mean (SEM). Statistical analysis was performed by means of one-way analysis of variance followed by the least significant difference test using SPSS 16.0 software (USA). A p value of < 0.05 was regarded as statistically significant, and < 0.01 was regarded as highly significant.

## Results

### Resveratrol improves the viability of HUVECs impaired by TNF-α

**Fig 1:**
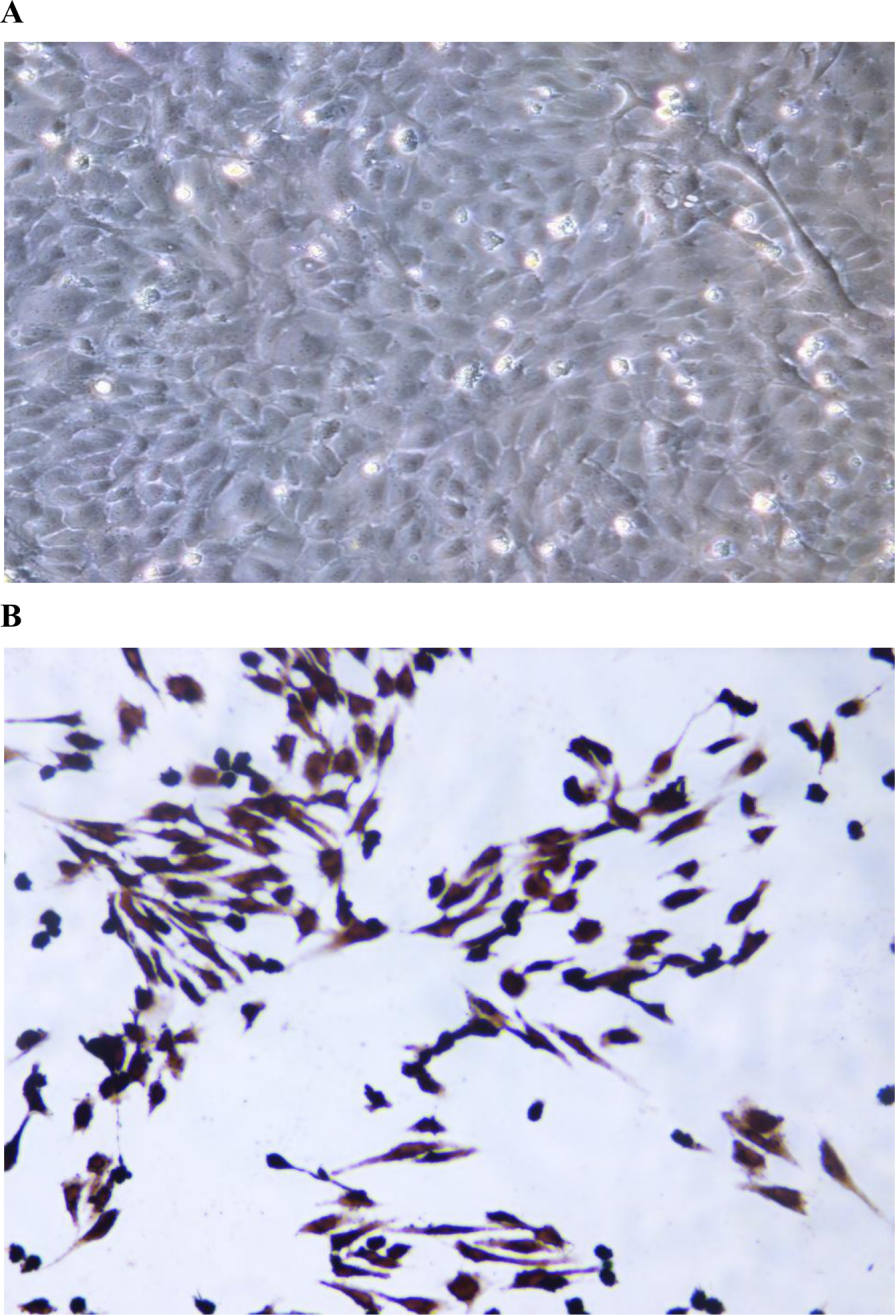
Primary HUVECs (×200). **(A)** The morphology of primary HUVECs was observed under an inverted, phase-contrast microscope. The cells were homogenous, closely apposed, large, flat, and polygonal with a cobblestone-shaped appearance. **(B)** The cytoplasm of the HUVECs was stained brown-red after incubation with a rabbit anti-factor VIII antigen by the immunocytochemical staining method. The purity of the cultured HUVECs was more than 95%.

**Fig 2:**
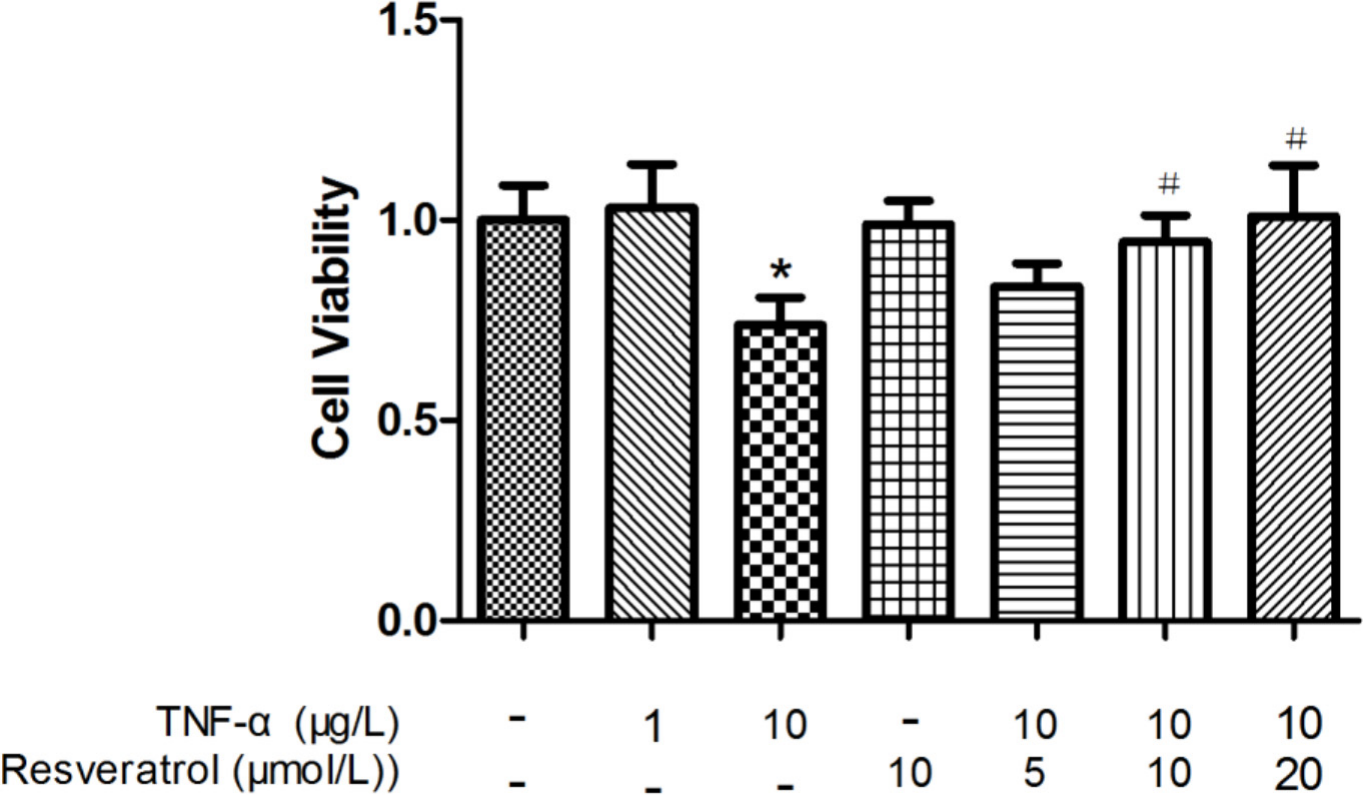
Resveratrol increases cell viability in TNF-α-treated HUVECs. Cell viability was assessed via the CCK-8 method. HUVECs were exposed to different concentrations of resveratrol (0, 5, 10, and 20 μM) before treatment with TNF-α (10 μg/L). Data are expressed as the mean ± SEM from six independent experiments, each carried out in triplicate. *p < 0.05 vs. control; #*p* < 0.05 vs. TNF-α alone.

HUVECs were grown as a confluent monolayer with cobblestone morphology (Fig 1A), and the cytoplasm was stained brownish red after incubation with an anti-VIII factor antigen (Fig 1B). As shown in Fig 2 and S1 Table, 10 μg/L, but not 1 μg/L, TNF-α induced a significant difference in cell viability, compared with the control group (*p < 0.05 vs. control). Therefore, we used 10 μg/L TNF-α as the proper dose to induce the inflammatory response. Since a high concentration of resveratrol (>30 μM) has been shown previously to impair cell viability [19], we treated HUVECs with resveratrol at concentrations less than 30 μM (0, 5, 10, and 20 μM) to investigate the effect of resveratrol on the viability of HUVECs. While 10 μM resveratrol did not have any significant effect on the viability of HUVECs, it greatly improved the survival of HUVECs impaired by TNF-α (#*p* < 0.05 vs. TNF-α alone). Similar results were observed with 20 μM resveratrol. These observations suggest that resveratrol improves the viability of HUVECs impaired by TNF-α treatment.

### Resveratrol attenuates the increased expression of CD40 triggered by TNF-α stimulation in HUVECs

**Fig 3:**
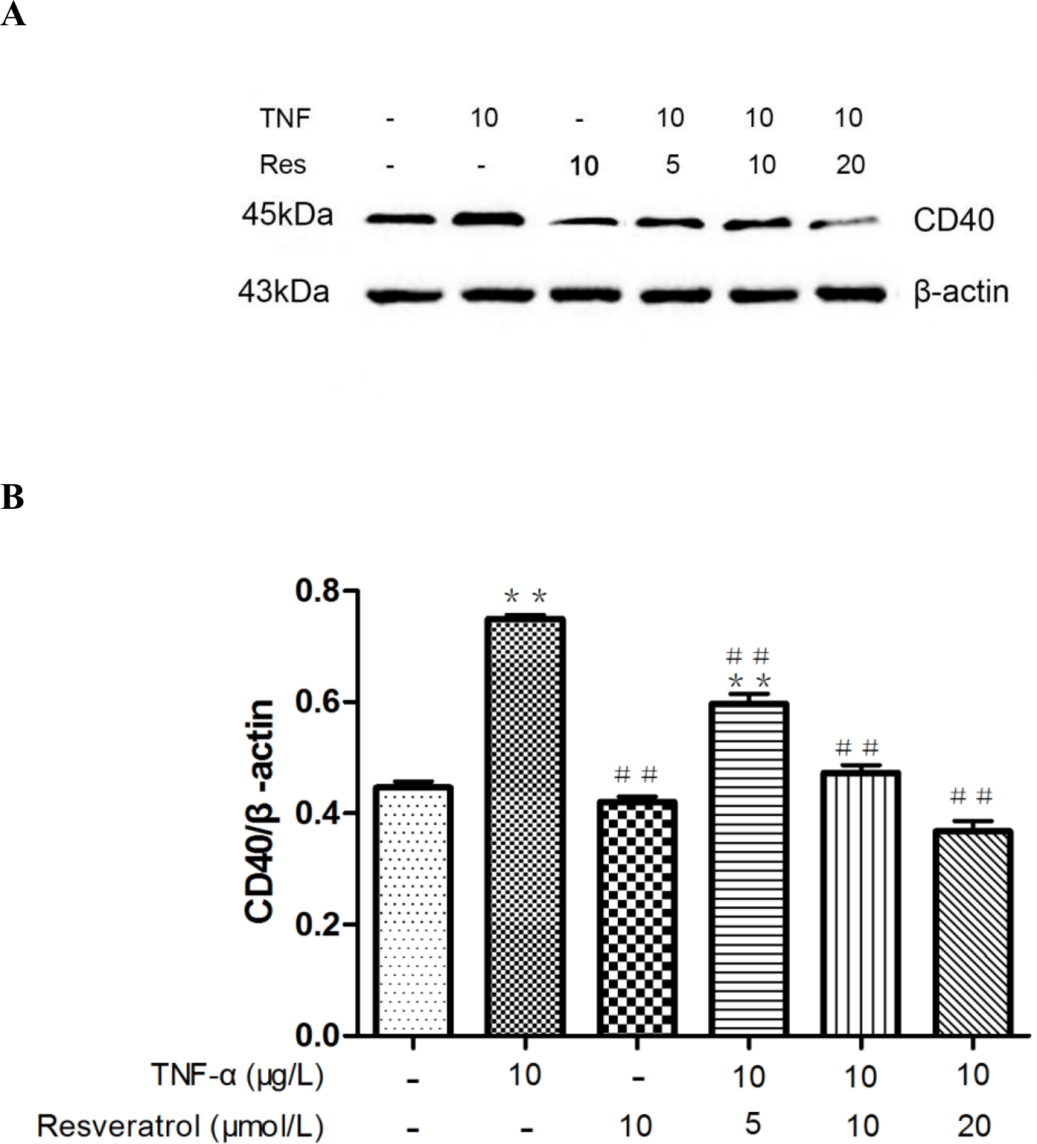
Resveratrol attenuates the TNF-α-induced CD40 expression in HUVECs. **(A)** Resveratrol was added at different concentrations as indicated to TNF-α-stimulated HUVECs, and CD40 expression was determined by western blot. The representative data from three independent assays are shown. **(B)** Densitometric analysis of the blots shown in A. The data are expressed as the mean ± SEM from three independent experiments, each carried out in triplicate. **p < 0.01 vs. control; ##*p* < 0.05 vs. TNF-α alone.

Increased expression of the CD40 receptor activated by CD40L has been shown to alter the expression levels of specific adhesion molecules, thus promoting the inflammatory response [20]. In addition, increased expression of CD40 also has been shown to be involved in the TNF-α-induced inflammatory response [21]. To determine whether resveratrol alters TNF-α-induced CD40 expression in HUVECs, cells were preincubated with different concentrations of resveratrol prior to TNF-α stimulation, followed by western blot analysis to determine the protein levels of CD40 (Fig 3A). While TNF-α significantly induced the expression of CD40 as expected (**p < 0.01 vs. control), this induction was downregulated by resveratrol in a dose-dependent fashion (##*p* < 0.01 vs. TNF-α alone), although 10 μM resveratrol did not have any significant effect on CD40 expression at baseline. Thus, we conclude that resveratrol treatment attenuates the increased expression of CD40 triggered by TNF-α stimulation.

### Resveratrol suppresses TNF-α-triggered CD40 expression via activating SIRT1 in HUVECs

**Fig 4:**
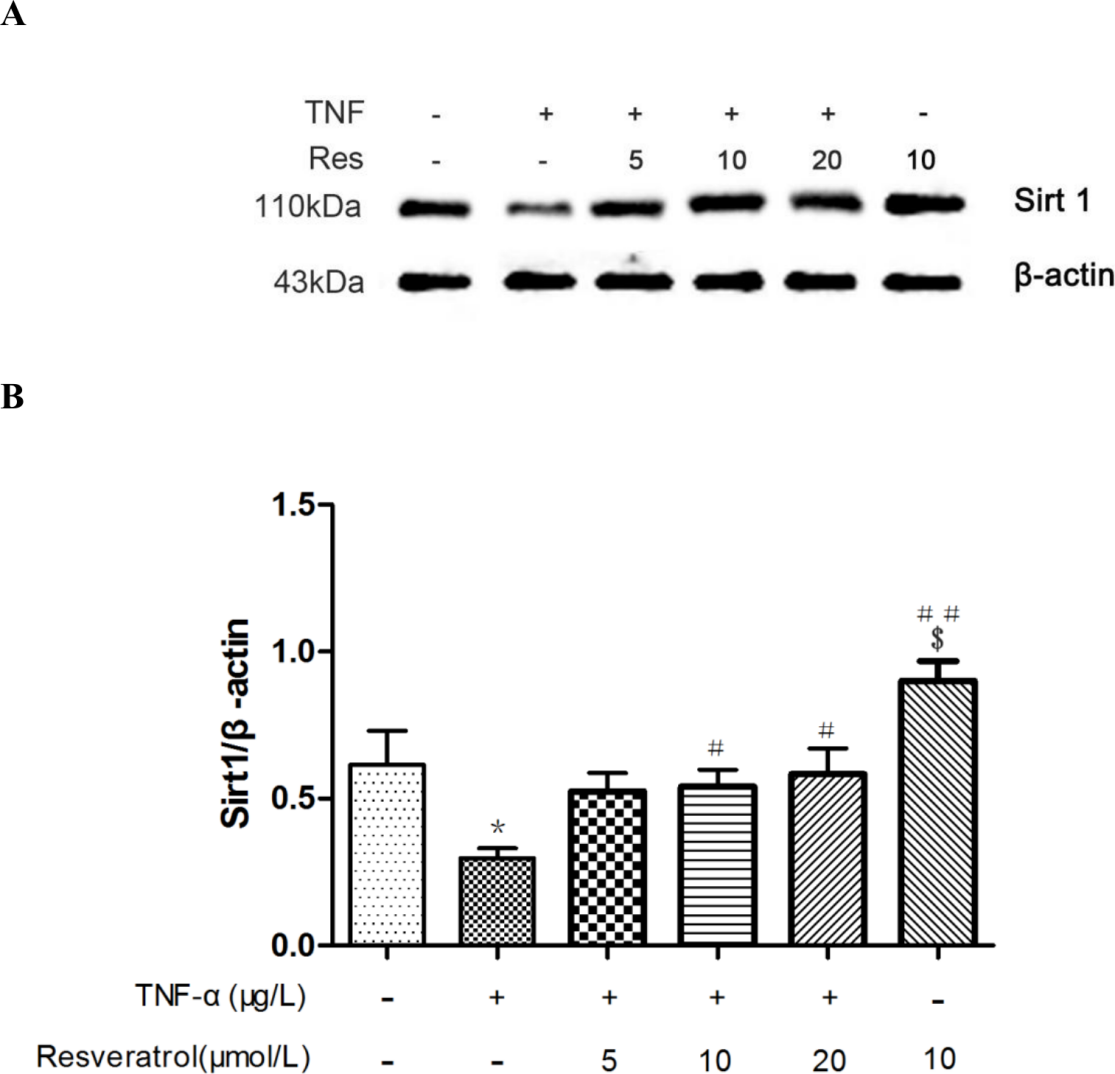
Resveratrol increases the expression of SIRT1 in TNF-α-treated HUVECs. HUVECs were either untreated (control), treated with TNF-α (10 μg/L) alone, resveratrol (10 μM) alone, or cotreated with TNF-α (10 μg/L) and different concentrations of resveratrol as indicated for 24 h. **(A)** Western blot analysis was carried out on the protein lysates purified from HUVECs of the above-mentioned groups. β-Actin was used as a loading control. **(B)** Densitometric analysis of the blots shown in A. The data are expressed as the mean ± SEM from three independent experiments, each carried out in triplicate. *p < 0.05 vs. control; #*p* < 0.05, ##*p* < 0.01 vs. TNF-α alone; $*p* < 0.05 vs. control.

A previous study has suggested that resveratrol activates SIRT1 [22]. Therefore, we analyzed the expression of SIRT1 in HUVECs incubated with different concentrations of resveratrol (0, 5, 10, and 20 μM) in the absence or presence of TNF-α. As shown in Fig 4 and S3 Table, western blot analysis showed that the protein levels of SIRT1 were significantly decreased upon stimulation with 10 μg/L TNF-α (*p < 0.05 vs. control). However, this decrease was significantly attenuated by 10 and 20 μM resveratrol treatment (#*p* < 0.05 vs. TNF-α alone). Intriguingly, 20 μM resveratrol treatment increased the expression of SIRT1 at baseline ($*p* < 0.05 vs. control). Thus, we conclude that resveratrol improves the expression of SIRT1, which was suppressed by TNF-α stimulation.

**Fig 5:**
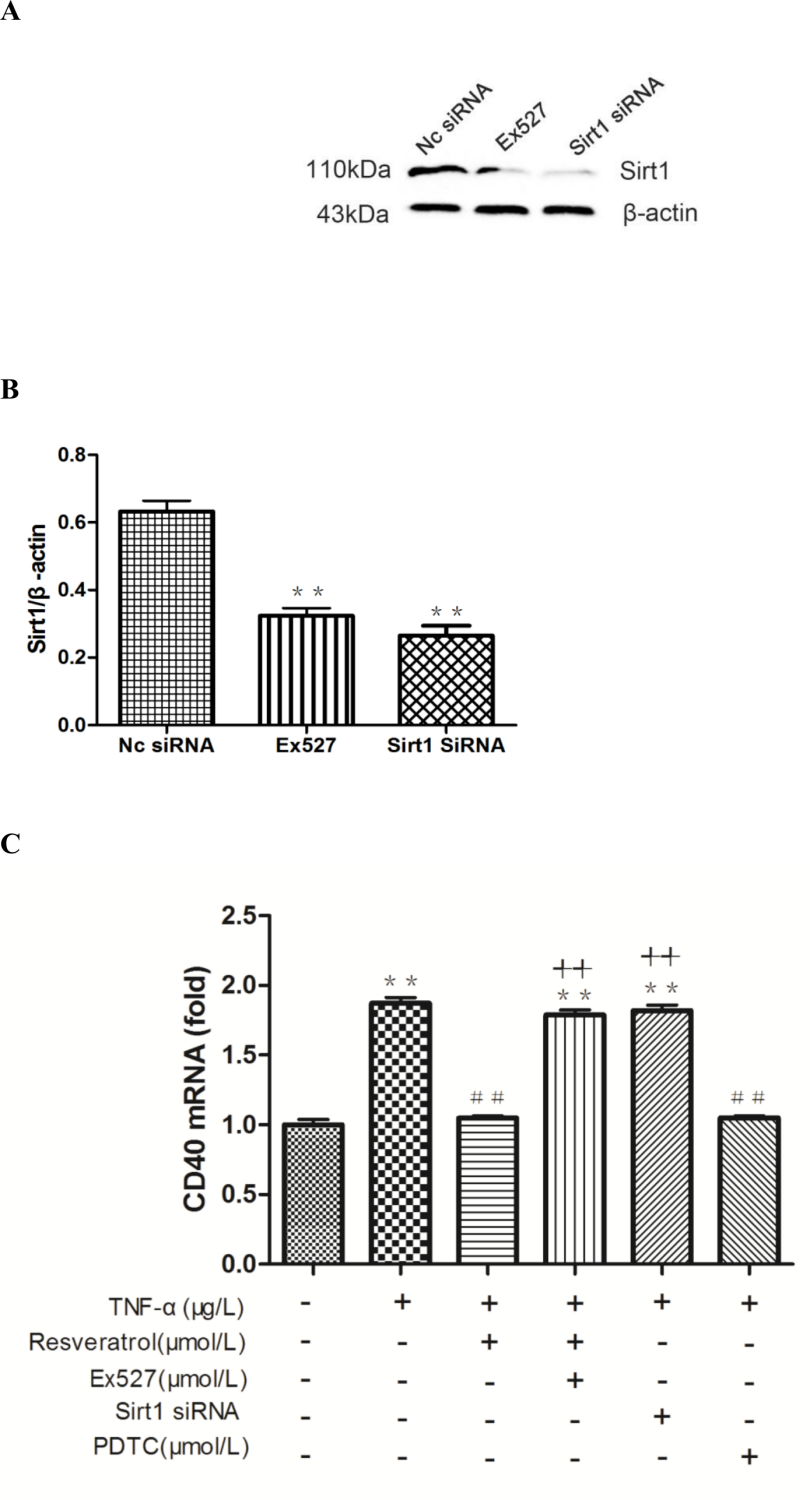
Inhibition of SIRT1 reverses the resveratrol-imposed suppression of CD40 expression in TNF-α-treated HUVECs. **(A)** HUVECs were either transfected with specific siRNA against SIRT1, Nc-siRNA (scrambled siRNA), or Ex527 with Lipofectamine for 24 h. Whole cell lysates were subjected to western blot analysis. **(B)** Densitometric analysis of the blots shown in A. **p < 0.05 vs. Nc-siRNA group. **(C)** RT-qPCR was performed as described in the Materials and Methods section. TNF-α, 10 μg/L; resveratrol, 10 μM; Ex527, 10 μM; PDTC, 20 μM. The results are expressed as the mean ± SEM from three independent assays, each carried out in triplicate. **p < 0.01 vs. control; ##*p* < 0.01 vs. TNF-α alone; ++*p* < 0.01 vs. TNF-α + resveratrol.

**Fig 6:**
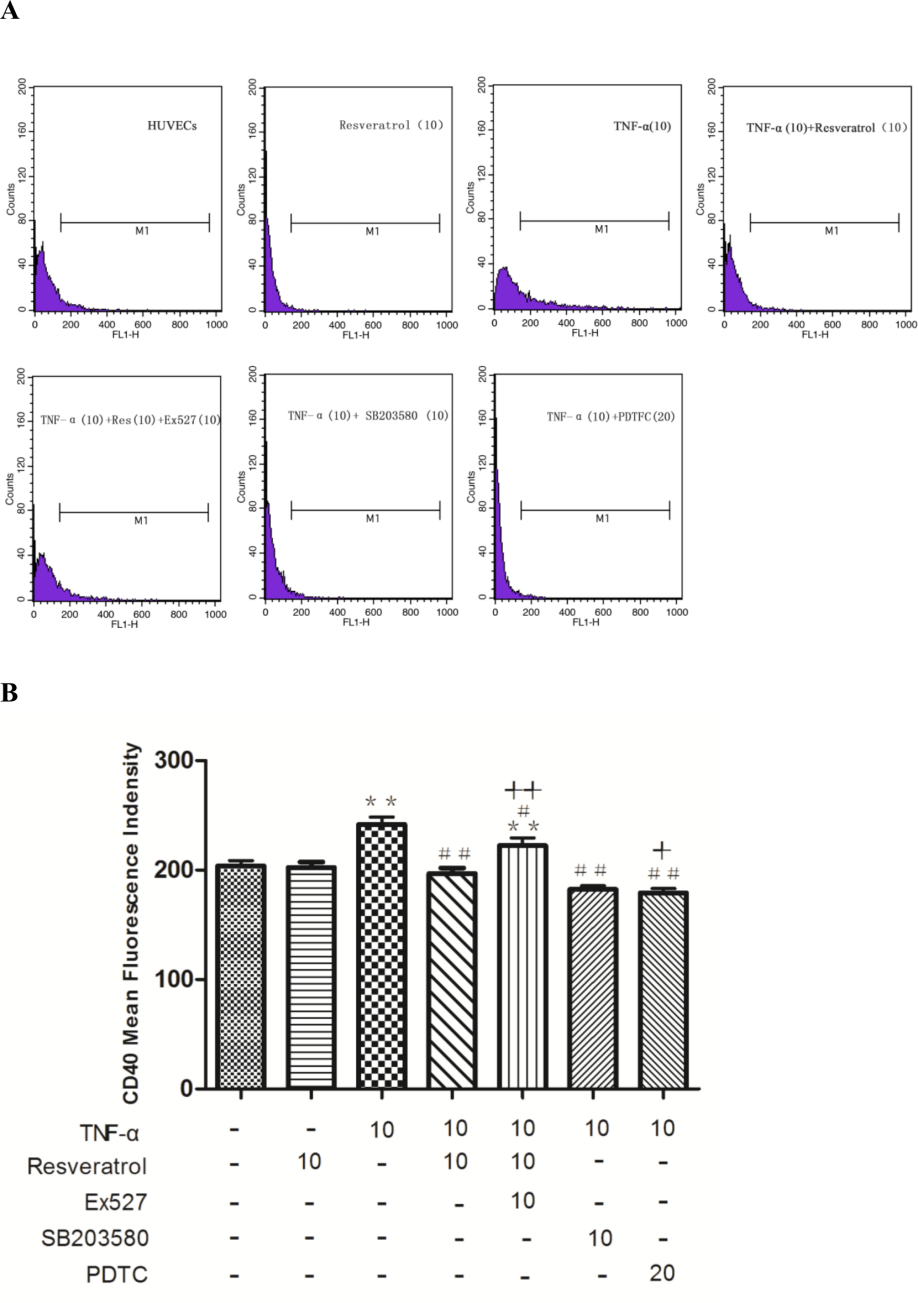
Inhibition of SIRT1 reduces the antagonistic effects of resveratrol on the TNF-α-induced increase in CD40 expression in HUVECs. HUVECs were pretreated with 10 μM resveratrol in the presence or absence of Ex527 before TNF-α stimulation. PDTC and SB203580 were also applied to explore the involvement of the respective signaling pathways. **(A)** Representative images of the flow cytometry experiment show each experimental condition and the cell percentages in each gate from independent experiments. More than 5 × 10^3^ cells were detected. **(B)** The mean fluorescence intensity of CD40 was calculated. **p < 0.01 vs. control; #*p* < 0.05 and ##*p* < 0.01 vs. TNF-α alone; +*p* < 0.05 and ++*p* < 0.01 vs. TNF-α + resveratrol.

As shown in Fig 3 and S2 Table, CD40 expression in HUVECs was decreased with increasing concentrations of resveratrol ranging from 5 μM to 20 μM. Next, we used a loss-of-function approach, i.e., RNA interference or the SIRT1 inhibitor Ex527 to knock down SIRT1 gene expression to explore whether SIRT1 is involved in the resveratrol-mediated expression of CD40. The HUVECs with transfected siRNA specifically targeting SIRT1 or treatment with Ex527 exhibited significantly decreased SIRT1 expression, and the knockdown efficiency was approximately 73.38 ± 0.8363% (S4 Table, S5 Table, Figs 5 and 6). The intracellular CD40 expression inhibited by resveratrol was significantly increased in response to SIRT1 knockdown or the SIRT1 inhibitor (++*p* < 0.01 vs. TNF-α + resveratrol).

**Fig 7:**
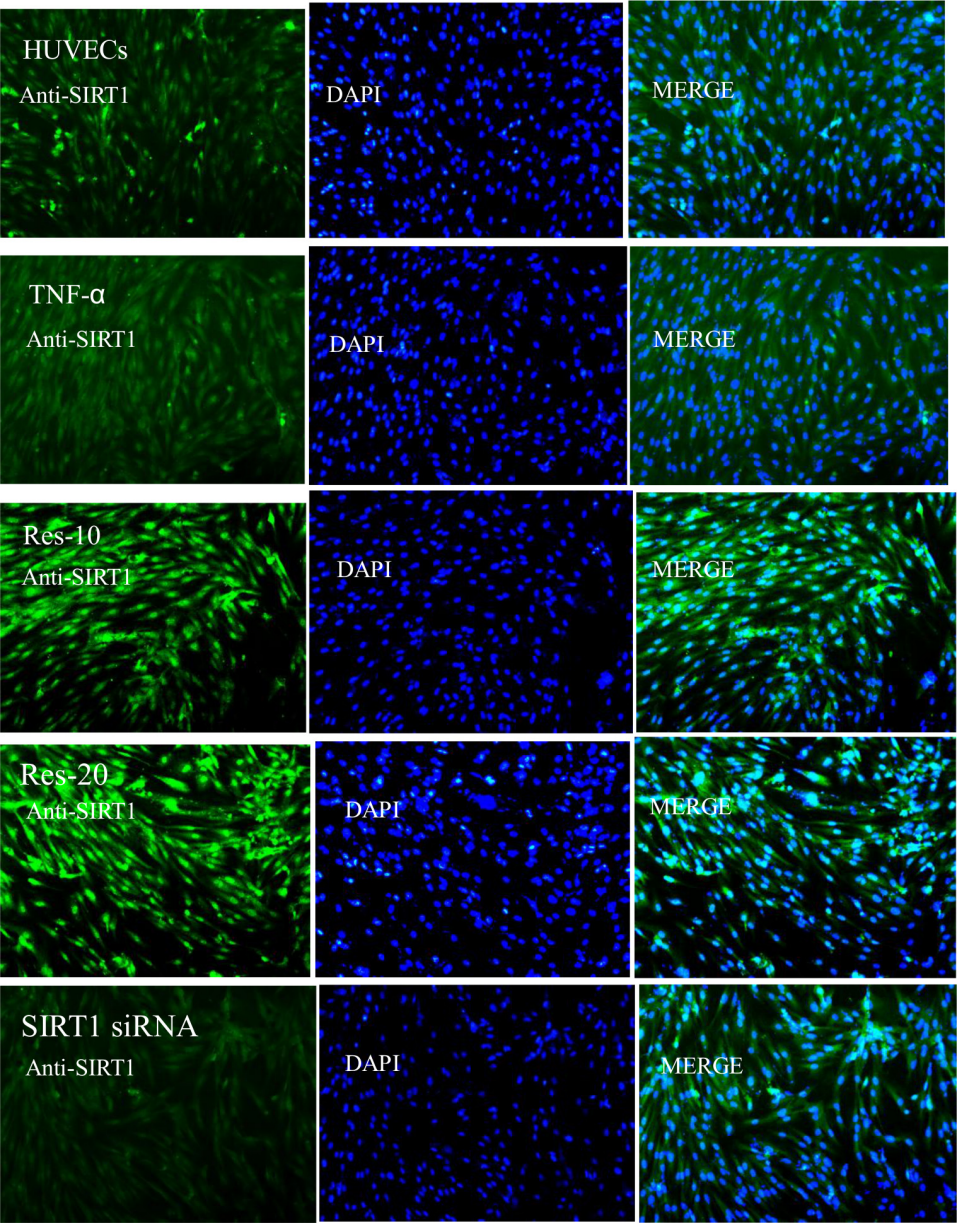
SIRT1 is localized in both the cytoplasm and the nucleus in HUVECs. SIRT1 was stained green with an anti-SIRT1 antibody. The nuclei were stained with DAPI, and the merge represents the combined image of SIRT1 fluorescence and nuclear staining. The cells were pretreated with different concentrations of resveratrol or transfected with SIRT1 siRNA, followed by TNF-α stimulation for 24 h. Representative images are shown to indicate the impact of resveratrol on the subcellular localization of SIRT1. The same results were obtained in three independent experiments. Magnification, 200×.

To further examine the subcellular location and expression of SIRT1 in HUVECs, cells were exposed to TNF-α (10 μg/L) for 24 h in the absence or presence of 10 or 20 μM resveratrol or transfected with SIRT1 siRNA, followed by immunostaining to detect SIRT1 expression. As shown in Fig 7, fluorescent puncta were significantly increased by resveratrol at 10 and 20 μM, but these increases were significantly attenuated by SIRT1 siRNA. As shown in Fig 7, immunofluorescence labeling with a specific antibody against SIRT1 demonstrated that SIRT1 is mainly localized in the nucleus but also is present in the cytoplasm of HUVECs.

### Resveratrol suppresses TNF-α-triggered ROS generation via activating SIRT1 in HUVECs

**Fig 8:**
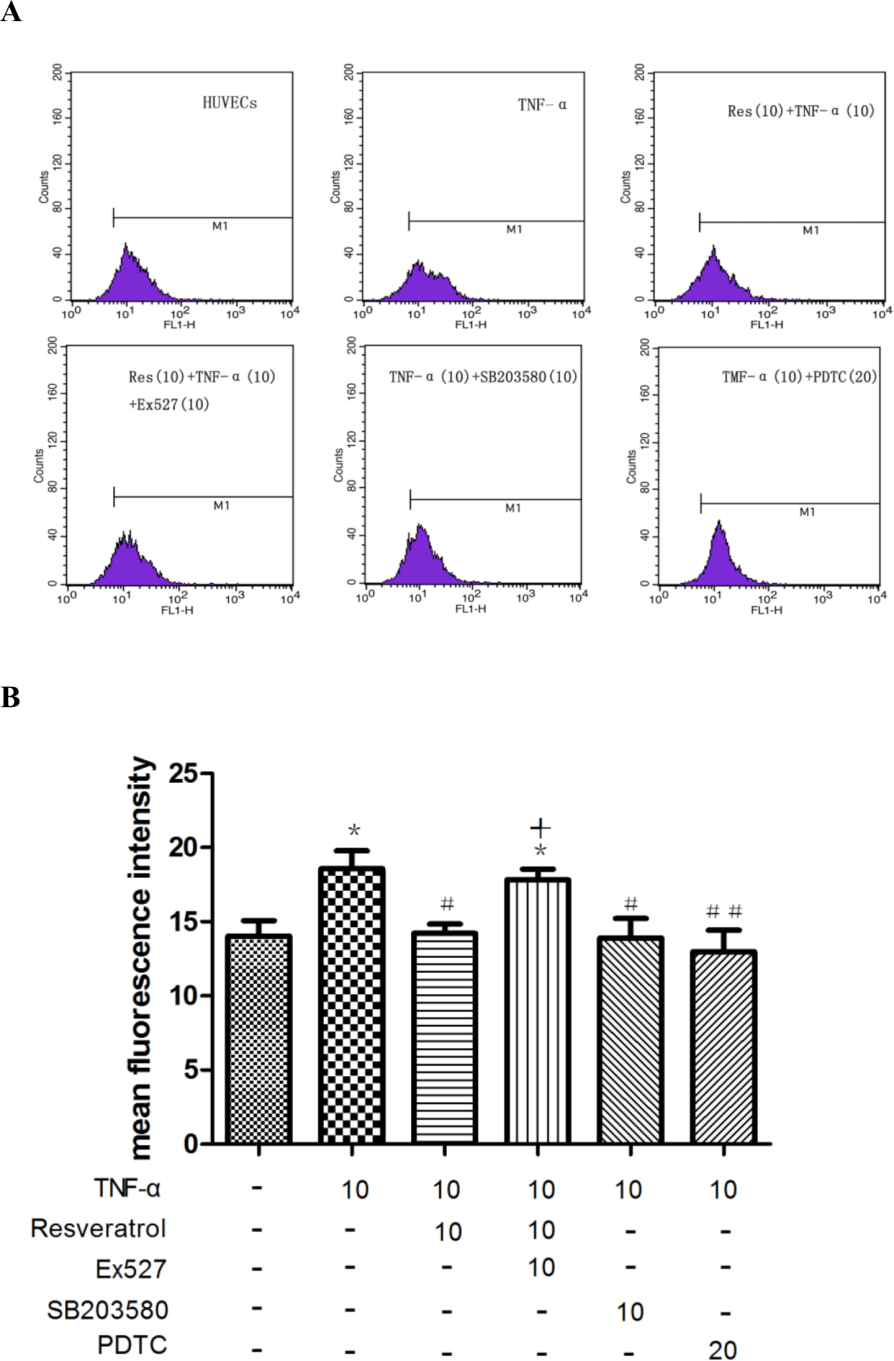
Inhibition of ROS production in TNF-α-treated HUVECs by resveratrol was compromised by suppression of SIRT1. **(A and B)** Cells were pretreated with resveratrol (10 μM) in the presence or absence of Ex527, PDTC, and SB203580 before TNF-α stimulation. The cells were stained with DCHF-DA to evaluate ROS generation. More than 1 × 10^4^ cells were analyzed by flow cytometry. *p < 0.05 vs. controls; #*p* < 0.05 and ##*p* < 0.01 vs. TNF-α alone; +*p* < 0.05 vs. TNF-α + resveratrol.

To investigate the anti-oxidant effect of resveratrol, we evaluated ROS generation in HUVECs after resveratrol treatment using DCFH-DA staining, and the mean fluorescence intensity was measured by flow cytometry. As shown in Fig 8 and S6 Table, TNF-α (10 μM) significantly increased ROS production (*p < 0.05 vs. control), which was substantially attenuated by resveratrol (10 μM) (#*p* < 0.05 vs. TNF-α alone). A similar inhibitory effect was observed by using PDTC, an inhibitor of NF-κB, and SB203580, a MAPK inhibitor. We also found that cotreatment with Ex527, an inhibitor of SIRT1, impaired the effectiveness of resveratrol (+*p* < 0.05 vs. TNF-α + resveratrol) in reducing ROS production. Taken together, we argue that resveratrol suppresses TNF-α-triggered ROS generation in HUVECs via potentiating the activity of SIRT1 and that both NF-κB and MAPK signaling are involved in mediating TNF-α-induced ROS generation.

### Resveratrol inhibits the induction of NF-κB activity by TNF-α via activating SIRT1

**Fig 9:**
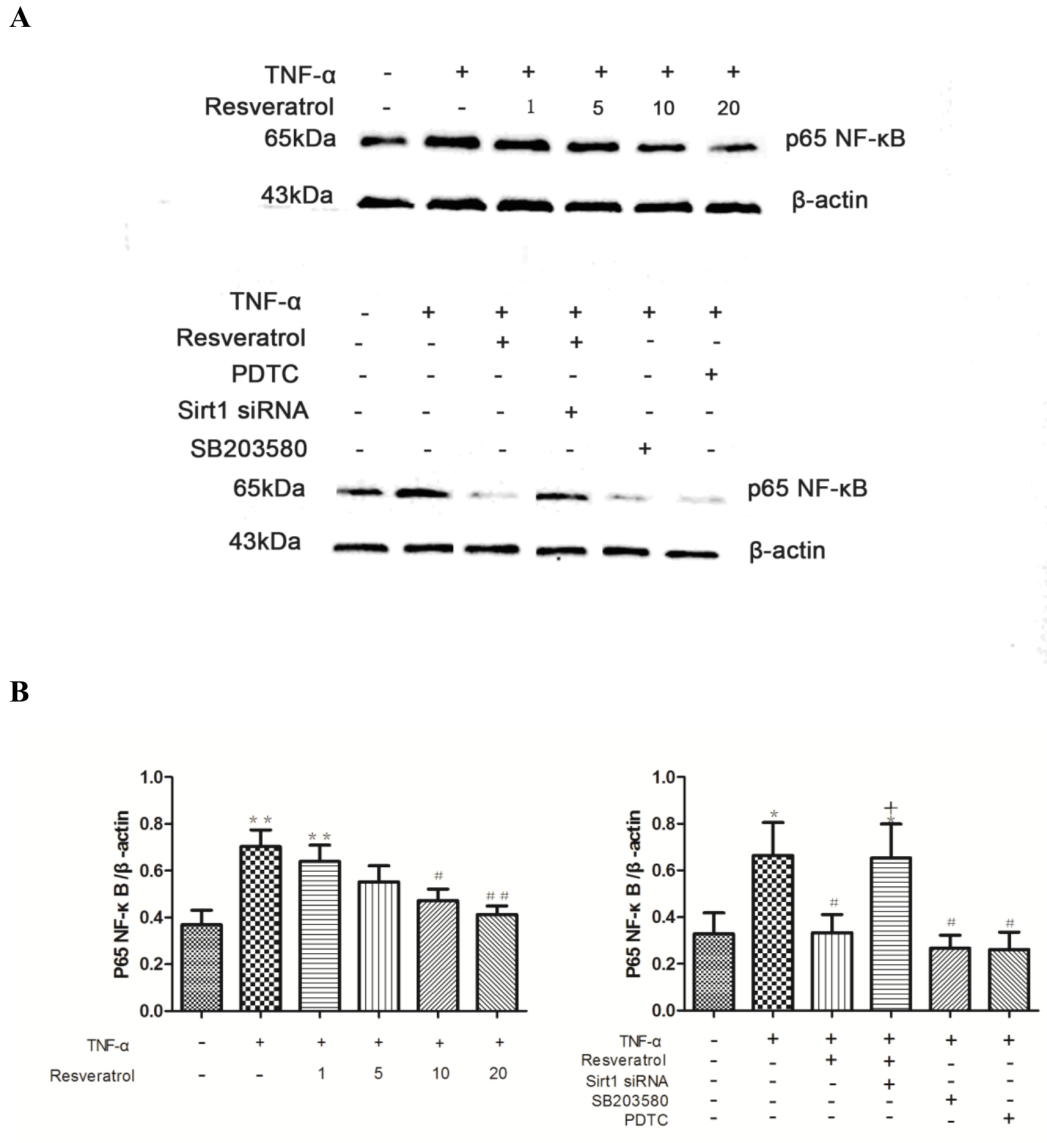
Depletion of SIRT1 ameliorates the inhibitory effect of resveratrol on the expression of NF-κB p65 in TNF-α-treated in HUVECs. **(A)** HUVECs were transfected with SIRT1-specific siRNA or Nc-siRNA, followed by stimulation with 10 μg/L TNF-α with or without resveratrol (0–20 μM) as indicated. The expression of NF-κB p65 was evaluated by western blot performed on the protein extracts from the cells of different groups. SB203580 (10 μM) or PDTC (20 μM) was applied to explore the mechanism. **(B)** Densitometric analysis of the blots shown in A. The results are expressed as the mean ± SEM from three independent experiments. **(C)** HUVECs were transiently transfected with a reporter plasmid containing five copies of an NF-κB response element that drives transcription of the luciferase reporter gene and siRNA targeting SIRT1. On the next day, transfected cells were pretreated with resveratrol (20 μM), SB203580 (10 μM), or PDTC (20 μM). The cells were stimulated with the indicated concentrations of TNF-α, and the cell lysates were collected and analyzed for luciferase activity. **(D)** The mRNA levels of NF-κB were analyzed by the PCR method. **p < 0.01 vs. control; #*p* < 0.05 and ##*p* < 0.01 vs. TNF-α alone; +*p* < 0.05 and ++*p* < 0.01 vs. TNF-α + resveratrol.

NF-κB signaling has been reported previously to be involved in mediating the cellular inflammatory response [23]. To probe if resveratrol has any effect on TNF-α-induced NF-κB signaling in HUVECs, western blot analysis was performed on protein lysates purified from HUVECs with different treatments to evaluate the expression of p65. As shown in Fig 9A and 9B, TNF-α treatment significantly increased the expression of p65 (**p < 0.01 vs. control), which was greatly suppressed by resveratrol at concentrations of 10 and 20 μM (#*p* < 0.05, ##*p* < 0.01 vs. TNF-α alone). As expected, PDTC, an inhibitor of NF-κB, substantially inhibited the expression of p65. In addition, SB203580, a MAPK inhibitor, also exhibited a similar inhibitory impact on p65 expression as PDTC.

Next, to investigate the potential link between the suppression of NF-κB and the activation of SIRT1 by resveratrol, HUVECs were transfected with SIRT1 siRNA 24 h prior to the experiments, followed by western blot analysis, PCR, and the luciferase reporter assay to detect the expression levels of p65. As shown in Fig 9B (right panel), 9C, and 9D, depletion of SIRT1 by siRNA significantly increased the expression of p65. Therefore, we conclude that resveratrol suppresses the TNF-α-induced expression of p65 at least in part via activating SIRT1.

### Resveratrol attenuates TNF-α-induced p38 phosphorylation via activating SIRT1

**Fig 10:**
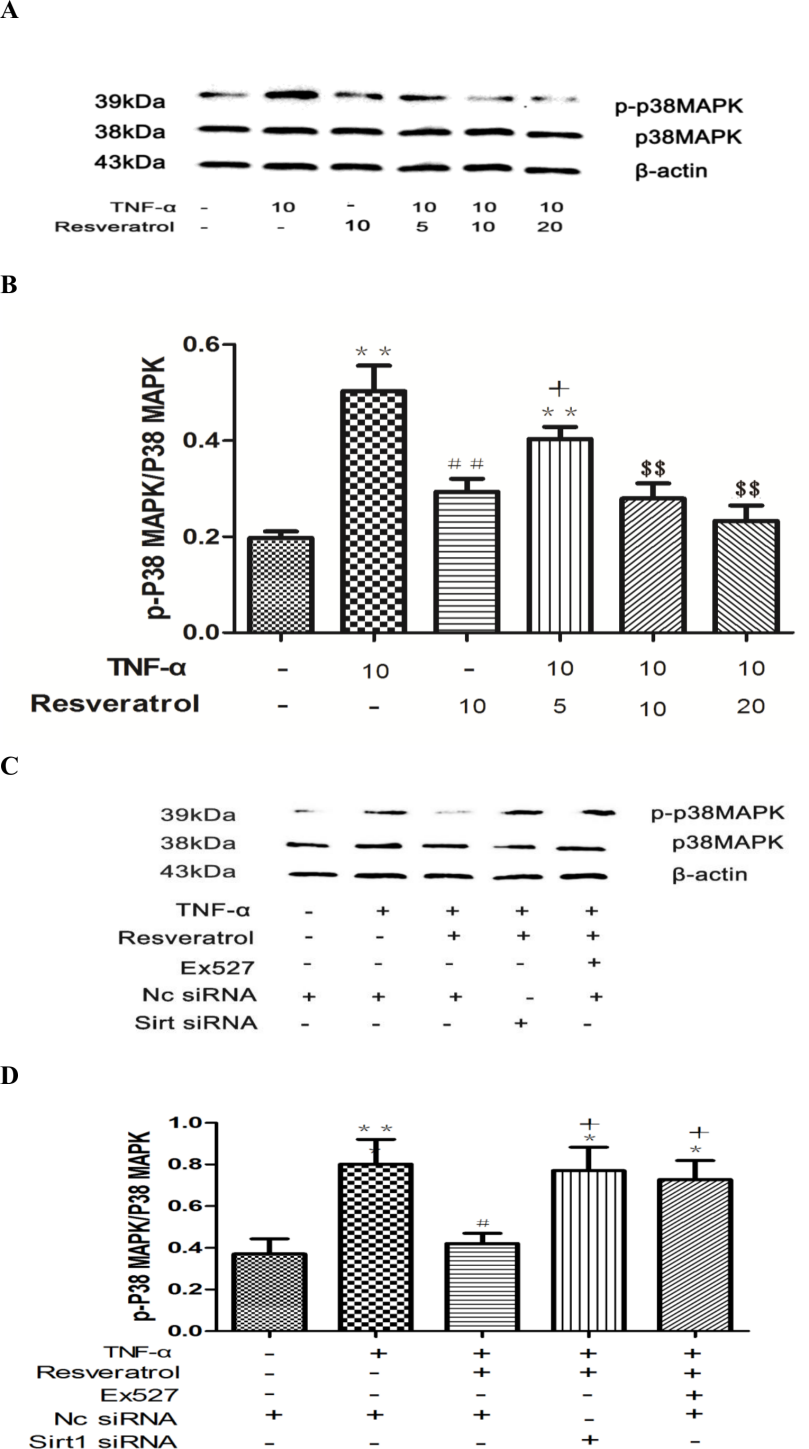
Inhibition of SIRT1 ameliorates the repressive effect of resveratrol on phospho-p38 MAPK in TNF-α-treated in HUVECs. **(A)** HUVECs were treated with different concentrations of resveratrol (0, 5, 10, and 20 μM) for 4 h, and the extracted whole cell lysates were subjected to western blot analysis using antibodies against phospho-p38 MAPK and total p38 MAPK, respectively. The phospho-p38/total p38 ratio was calculated. **(B)** Densitometric analysis of the blots shown in A. Data were compiled from three independent experiments and are expressed as the mean + SEM. **p < 0.01 vs. control; ##*p* < 0.01 and $ $*p* < 0.01 vs. TNF-α alone; +*p* < 0.05 vs. TNF-α + resveratrol. **(C)** HUVECs were pretreated with Ex527 (10 μM) or transfected with SIRT1 siRNA, followed by cotreatment with resveratrol and TNF-α as indicated. Western blot analysis was performed as described in A. **(D)** Densitometric analysis of the blots shown in C. Data were compiled from three independent experiments and are expressed as the mean + SEM. *p < 0.05 and **p < 0.01 vs. control; #*p* < 0.05 vs. TNF-α alone; +*p* < 0.05 vs. TNF-α + resveratrol.

ERK, p38 MAPK, and JNK are all members of the MAPK family. Since SB203580, a MAPK inhibitor, exhibited an inhibitory impact on p65 expression (Fig 9 and S7 Table), we next explored the possible involvement of MAPK pathways in resveratrol-mediated protection against inflammation and oxidative stress. Flow cytometry analysis showed that CD40 expression and ROS generation induced by TNF-α were both inhibited by SB203580 (Fig 8A and 8B). Since phosphorylation is a post-translational modification that regulates the activity of MAPKs [24], we next examined the effect of resveratrol on phospho-p38 MAPK. TNF-α alone increased the phospho-p38 MAPK expression compared with the control, but this increase was significantly attenuated by pretreatment with resveratrol in a dose-dependent manner (Fig 10A and 10B, $ $*p* < 0.01 vs. TNF-α alone). To explore whether SIRT1 is involved in resveratrol-suppressed p38 MAPK phosphorylation, we inhibited SIRT1 by both siRNA-knockdown and Ex527 in HUVECs during resveratrol treatment in the presence of TNF-α. Inhibition of SIRT1 significantly reversed the repressive effects of resveratrol on p38 phosphorylation (Fig 10C and 10D, +*p* < 0.05 vs. TNF-α + Nc-siRNA). Collectively, we conclude that resveratrol attenuates TNF-α-induced p38 phosphorylation via activating SIRT1.

### p38 MAPK acts upstream of the NF-κB signaling pathway

As mentioned above, the NF-κB pathway plays a critical role in the inflammatory response. Given that the increased CD40 expression and ROS production elicited by TNF-α stimulation were significantly diminished by treatment with PDTC, a NF-κB signaling inhibitor (in S4 Table, S5 Table, Figs 5C, 6 and 8), we next asked whether there is any functional correlation between p38 MAPK and NF-κB signaling. As shown in Fig 9 and S8 Table, SB203580 treatment significantly inhibited the expression of p65 induced by TNF-α to the same level as PDTC. Thus, p38 MAPK may act as an upstream activator of the NF-κB p65 pathway.

## Discussion

In this study, we focused on the protective effects of resveratrol against inflammatory and oxidative injury in HUVECs induced by TNF-α. Given the important roles of the CD40/CD40L axis in the inflammatory response and ROS production in oxidative stress during the pathological process of atherosclerosis, we explored the mechanisms by which resveratrol reduces CD40 expression and ROS generation induced by TNF-α. Based on previous findings that only low-to-moderate doses of resveratrol are able to attenuate ROS exposure while high doses induce cytotoxicity, the latter of which is caused by mitochondrial dysfunction [25], in the present study, we used resveratrol in the concentration range of 0–20 μM. By examining the effect of resveratrol on cell viability decreased by TNF-α, we determined the effective concentration of resveratrol to be 10–20 μM. Since CD40 and CD40L are both transmembrane proteins structurally related to the cytokine TNF-α, we stimulated cells with TNF-α to mimic the inflammatory microenvironment in vascular tissues.

Resveratrol is a subtype of phytoalexins that protects against pathological processes through suppressing elevated levels of proinflammatory cytokines in macrophages. For instance, it has been shown that the CD40/CD40L levels in rats with hypercholesterolemia are downregulated by trans-resveratrol administration [26]. Another study has suggested that resveratrol decreases the CD40 expression in dendritic cells to regulate tolerogenic effects [27]. In line with these reports, we found that resveratrol attenuated CD40 expression induced by TNF-α in HUVECs. The underlying mechanisms by which resveratrol exerts its inhibitory effects on CD40 expression have been attempted previously by a number of groups. For example, Takizawa *et al*. have found that the expression levels of eNOS and SIRT1 are upregulated in HUVECs treated repeatedly with 1 mM resveratrol for 6 days. Moreover, Cantó *et al*. have reported that resveratrol activates SIRT1 by the cAMP–Epac1–AMPK pathway [28], suggesting that eNOS and SIRT1 are important mediators for resveratrol-triggered effects on the cardiovascular system [29]. In addition, Yang *et al*. [30] and Lin *et al*. [31] have reported a functional link between SIRT1 activity and CD40 expression in CRL-1730 endothelial cells and 3T3-L1 adipocytes, respectively. In their findings, SIRT1 exerts its inhibitory effects on CD40 expression by deacetylating the RelA/p65 subunit of NF-κB at lysine 310. In accordance with the above findings, our work provided evidence that the protein levels of CD40 and the p65 subunit were downregulated by resveratrol and that these effects were reversed by inhibiting SIRT1 activity, suggesting that the anti-CD40 effect of resveratrol is attributable, at least to some extent, to the SIRT1-mediated suppression of the NF-κB pathway.

Resveratrol is also known as a robust scavenger of ROS. In normal bodies, there is a balance between ROS production and clearance. However, chronic inflammation can induce continuous production of high levels of ROS. Therefore, the uptake of exogenous antioxidants to clear ROS is believed to interfere with oxidative stress in a beneficial manner. Previous studies have shown that resveratrol directly attenuates the generation of superoxide (O_2_-), hydroxide (OH-), and peroxynitrite (ONOO-) radicals [32, 33]. Consistent with the above findings, our study suggested that resveratrol is able to reduce the production of ROS in HUVECs induced by TNF-α treatment. However, the mechanism of resveratrol on antagonizing oxidative stress is not fully understood. Some studies have reported that resveratrol may modulate the expression and activity of antioxidant enzymes through regulating the activity of transcription factors, including nuclear factor E2-related factor 2 [34], activator protein (AP-1), FOXO, and SP-1 [35–37]; and other studies have suggested that resveratrol supplementation conveys resistance to oxidative stress by diminishing oxidative mitochondrial membrane damage [38]. SIRT1 regulates the activity of multiple downstream targets such as PGC-1, Nrf-2, AP-1, FOXO, and SP-1, subsequently influencing the production of ROS. In addition, Zhang *et al*. have shown that oxidative stress induced by balloon injury could be attenuated by resveratrol in the rat carotid artery through altering the activity of the NF-κB pathway [39]. Consistent with these studies, our work revealed that SIRT1 siRNA, Ex527, or PDTC offsets the inhibitory effects of resveratrol on ROS production, supporting the notion that both SIRT1 and the NF-κB signaling cascade are the major regulatory components in the resveratrol-mediated protection against oxidative stress.

There is evidence that TNF-α promotes p38 MAPK phosphorylation and triggers the activation and translocation of NF-κB [40]. Ghisays *et al*. have reported that the N-terminal domain of SIRT1 promotes its physical association with its substrate NF-κB p65 in the nucleus [41]. According to our results, SIRT1 was located in both the cytoplasm and the nucleus. Thus, it is tempting to speculate that SIRT1 may mediate both p38 MAPK and NF-κB signaling pathways in a cell type-dependent fashion. Previous studies have suggested that phospho-p38 MAPK expression is downregulated by resveratrol, which has been shown to ameliorate diabetes-induced cardiac dysfunction [42], interleukin-6 release in PC12 cells [43], and oxidized low-density lipoprotein-induced macrophage apoptosis [44]. Our results confirmed that phospho-p38 MAPK expression induced by TNF-α could be reduced by resveratrol in a dose-dependent manner. Moreover, this inhibitory effect was reversed by inhibition of SIRT1 activity achieved by siRNA knockdown or treatment with its inhibitor Ex527, suggesting that SIRT1 mediates p38 phosphorylation. Interestingly, our study also found that the release of free nuclear NF-κB p65 was inhibited by SB203580, thus explaining why SB203580 exerted the same inhibitory effect as PDTC on CD40 expression and ROS production.

Taken together, our data provide a molecular basis underpinning the protective effect of resveratrol on inflammation and oxidative injury in HUVECs induced by TNF-α. By activating SIRT1, resveratrol protects cells from damage caused by inflammatory factors. TNF-α-induced NF-κB p65 expression and p38 MAPK phosphorylation were attenuated by SIRT1 activation triggered by resveratrol treatment. According to the subcellular distribution of SIRT1, SIRT1 may regulate phospho-p38 MAPK in the cytoplasm and p65 expression in the nucleus. As phospho-p38 MAPK promotes the translocation of p65, the effects of resveratrol on CD40 expression and ROS generation induced by TNF-α in HUVECs were either through the direct interaction of SIRT1 with NF-κB p65 or p38 MAPK-mediated arrest of p65. To the best of our knowledge, our study demonstrated for the first time that SIRT1-mediated inhibition of phospho-p38 MAPK and NF-κB p65 expression may be common pathways for resveratrol to regulate CD40 expression and ROS generation. These two pathways may also serve as a common bridge between the CD40/CD40L axis and ROS generation. However, whether there is a direct functional interaction between the CD40/CD40L axis and ROS generation still needs to be elucidated.

## Conclusions

In conclusion, CD40 expression and ROS generation are upregulated in HUVECs stimulated with TNF-α, and these effects are attenuated by resveratrol treatment. Resveratrol also increases the expression of SIRT1, which contributes to the inhibition of phospho-p38 MAPK in the cytoplasm and the reduced activity of NF-κB p65 in the nucleus. Thus, our work provides a molecular basis for resveratrol to be a potential therapeutic for atherosclerosis in the clinic.

## Supporting Information

S1 Table Cell viability assay in Fig 2. (PDF)

S2 Table Intracellular CD40 protein expression in HUVECs in Fig 3B. (PDF)

S3 Table WB analysis of SIRT1 in Fig 4B. (PDF)

S4 Table WB of SIRT1 expression and PCR analysis of CD40 expression in Fig 5B and 5C. (PDF)

S5 Table Flow cytometry analysis of CD40 expression in Fig 6B. (PDF)

S6 Table Flow cytometry of ROS production in.Fig 8B. (PDF)

S7 Table WB analysis of NF-κB p65 in Fig 9B. (PDF)

S8 Table Luciferase assay of NF-κB p65 in Fig 9C and PCR assay of NF-κB p65 in Fig 9D. (PDF)

S9 Table Expression of phospho-p38 MAPK in Fig 10. (PDF)
