# Supplementary material for: Resveratrol Protects against TNF-α-Induced Injury in Human Umbilical Endothelial Cells through Promoting Sirtuin-1-Induced Repression of NF-KB and p38 MAPK
Source: PLoS One. 2016 Jan 22;11(1):e0147034. doi: 10.1371/journal.pone.0147034 (PMC4723256; doi:10.1371/journal.pone.0147034)
Supplement: S1 Table — (PDF) [file pone.0147034.s001.pdf]

CKK-8

NC	TNF 1	TNF10	RES 10	TNF 10+RES 5	TNF 10+RES 10
0.8955	1.0034	0.8202	1.0033	0.9117	0.9558
0.9153	1.1577	0.8287	0.9154	0.8827	0.9383
1.0367	1.175	0.6962	1.0365	0.7956	0.8583
0.9718	0.925	0.7253	0.9618	0.7878	1.0581
1.0706	0.9686	0.6794	1.0705	0.8575	0.905
1.1102	0.9507	0.6781	0.9507	0.7684	0.9567

TNF 10+RES 20
1.0776
1.0176
1.1226
0.869
1.1299
0.836
