# Supplementary material for: Resveratrol Protects against TNF-α-Induced Injury in Human Umbilical Endothelial Cells through Promoting Sirtuin-1-Induced Repression of NF-KB and p38 MAPK
Source: PLoS One. 2016 Jan 22;11(1):e0147034. doi: 10.1371/journal.pone.0147034 (PMC4723256; doi:10.1371/journal.pone.0147034)
Supplement: S2 Table — (PDF) [file pone.0147034.s002.pdf]

WB CD40/β -actin

NC	TNF 10	Res 10	TNF 10+ Res 5	TNF 10+ Res 10	TNF 10+ Res 20
0.452282	0.749543	0.402286	0.599623	0.457839	0.346492
0.461886	0.762698	0.422889	0.626615	0.500802	0.404343
0.42491	0.736447	0.436591	0.563841	0.460801	0.352909
