# Supplementary material for: Resveratrol Protects against TNF-α-Induced Injury in Human Umbilical Endothelial Cells through Promoting Sirtuin-1-Induced Repression of NF-KB and p38 MAPK
Source: PLoS One. 2016 Jan 22;11(1):e0147034. doi: 10.1371/journal.pone.0147034 (PMC4723256; doi:10.1371/journal.pone.0147034)
Supplement: S3 Table — (PDF) [file pone.0147034.s003.pdf]

WB SIRT1/β -actin

NC	TNF 10	TNF 10+Res 5	TNF 10+Res 10	TNF 10+Res 20
0.624909	0.315581	0.5985884	0.6074933	0.6363111
0.411992	0.228845	0.397836	0.4284619	0.4146054
0.809408	0.344474	0.5753917	0.5876023	0.6998357

Res 10

0.945624
0.766325
0.98849
