# Supplementary material for: Resveratrol Protects against TNF-α-Induced Injury in Human Umbilical Endothelial Cells through Promoting Sirtuin-1-Induced Repression of NF-KB and p38 MAPK
Source: PLoS One. 2016 Jan 22;11(1):e0147034. doi: 10.1371/journal.pone.0147034 (PMC4723256; doi:10.1371/journal.pone.0147034)
Supplement: S4 Table — (PDF) [file pone.0147034.s004.pdf]

WB SIRT1/β -actin

Nc siRNA	Ex527	Sirt1 SiRNA
0.680689	0.312711	0.278427
0.679687	0.363864	0.242992
0.62296	0.354496	0.338192

PCR CD40 mRNA

1.06839	1.892047	1.071059	1.794032	1.851245	1.051161
0.934637	1.931147	1.051931	1.848556	1.867515	1.07328
0.998554	1.792518	1.02496	1.723861	1.738971	1.02589
