# Supplementary material for: Resveratrol Protects against TNF-α-Induced Injury in Human Umbilical Endothelial Cells through Promoting Sirtuin-1-Induced Repression of NF-KB and p38 MAPK
Source: PLoS One. 2016 Jan 22;11(1):e0147034. doi: 10.1371/journal.pone.0147034 (PMC4723256; doi:10.1371/journal.pone.0147034)
Supplement: S5 Table — (PDF) [file pone.0147034.s005.pdf]

CD40 Mean Fluorescence Indensity

NC	Res 10	TNF 10	TNF 10+RES 10	TNF 10+RES 10 +Ex527	TNF 10+ SB203580
203.02	202.01	232.6	192.13	234.5	187.65
212.33	211.3	254.58	207.05	222.05	182.85
196	194	237.87	191.21	210.59	176.72

TNF 10+ PDTC	
	184.34
	181.49
	171.22
