# Supplementary material for: Resveratrol Protects against TNF-α-Induced Injury in Human Umbilical Endothelial Cells through Promoting Sirtuin-1-Induced Repression of NF-KB and p38 MAPK
Source: PLoS One. 2016 Jan 22;11(1):e0147034. doi: 10.1371/journal.pone.0147034 (PMC4723256; doi:10.1371/journal.pone.0147034)
Supplement: S6 Table — (PDF) [file pone.0147034.s006.pdf]

ROS fluorenscence

NC	TNF10	TNF10+RES10	TNF 10+RES 10+Ex527	TNF 10+ SB203580
13.42	16.17	12.97	16.68	14.23
16.06	19.54	14.63	17.62	16.01
12.59	19.99	15.03	19.16	11.46

TNF 10+PDTC	
	13.42
	15.23
	10.3
