# Supplementary material for: Resveratrol Protects against TNF-α-Induced Injury in Human Umbilical Endothelial Cells through Promoting Sirtuin-1-Induced Repression of NF-KB and p38 MAPK
Source: PLoS One. 2016 Jan 22;11(1):e0147034. doi: 10.1371/journal.pone.0147034 (PMC4723256; doi:10.1371/journal.pone.0147034)
Supplement: S7 Table — (PDF) [file pone.0147034.s007.pdf]

P65 NF-κ B /β -actin

NC	TNF 10	TNF 10+RES 1	TNF 10+RES 5
0.272557	0.775092	0.7573501	0.613762
0.48515	0.771989	0.6455999	0.627945
0.347965	0.562882	0.518055	0.4158206

P65 NF-κ B /β -actin

NC	TNF 10	TNF 10+RES 10	TNF 10+RES 10+SIRT1 siRNA
0.479581	0.7863	0.8150307	0.4144588
0.172042	0.382414	0.3655895	0.1788053
0.334914	0.823999	0.7817413	0.4076148

TNF 10+RES 10	TNF 10+RES 20
0.496295	0.479581
0.5423	0.403424
0.37737	0.354592

TNF 10+SB203580	TNF 10+PDTC
0.3108216	0.39003
0.1563276	0.1328699
0.334623	0.2601321
