# Supplementary material for: Resveratrol Protects against TNF-α-Induced Injury in Human Umbilical Endothelial Cells through Promoting Sirtuin-1-Induced Repression of NF-KB and p38 MAPK
Source: PLoS One. 2016 Jan 22;11(1):e0147034. doi: 10.1371/journal.pone.0147034 (PMC4723256; doi:10.1371/journal.pone.0147034)
Supplement: S8 Table — (PDF) [file pone.0147034.s008.pdf]

NF-kB reporter luciferace

NC	TNF 10	TNF 10+RES 10	TNF 10+PDTC
1	5.481	1.29	1.15
1.12	4.676	1.31	1.26
0.99	5.474	1.12	1

NF-kB mRNA

NC	TNF 10	TNF 10+RES 10	TNF 10+RES 10 +SIRT1 siRNA
1.054728	1.985285	1.225479	1.198641
1.014439	2.040235	1.205899	1.226766
0.934617	1.972851	1.333407	1.168349

TNF 10+RES 10+SIRT1 siRNA
2.45
2.53
2.44

TNF 10+SB203580	TNF 10+PDTC
1.524568	1.449792
1.472511	1.506864
1.479223	1.467492
