# Supplementary material for: Resveratrol Protects against TNF-α-Induced Injury in Human Umbilical Endothelial Cells through Promoting Sirtuin-1-Induced Repression of NF-KB and p38 MAPK
Source: PLoS One. 2016 Jan 22;11(1):e0147034. doi: 10.1371/journal.pone.0147034 (PMC4723256; doi:10.1371/journal.pone.0147034)
Supplement: S9 Table — (PDF) [file pone.0147034.s009.pdf]

p-P38MAPK/P38MAPK

NC	TNF 10	RES 10	TNF 10+RES 5
0.184277	0.415267	0.2409987	0.3590307
0.224045	0.493684	0.330672	0.4453008
0.183644	0.598636	0.3084758	0.4049903

p-P38MAPK/P38MAPK

NC	TNF 10	TNF 10+RES 10	TNF 10+RES 10+SIRT1 siRNA
0.441771	0.981024	0.5114255	0.9420932
0.444859	0.847871	0.4066443	0.8102554
0.224444	0.574118	0.3429091	0.5601386

TNF 10+RES 10	TNF 10+RES 20
0.2589252	0.1963356
0.3408348	0.2966679
0.2381552	0.2055123

TNF 10+RES 10+Ex527
0.8483571
0.7859483
0.5482922
